# Transcriptional regulation of main metabolic pathways of *cyoA*, *cydB*, *fnr*, and *fur *gene knockout *Escherichia coli *in C-limited and N-limited aerobic continuous cultures

**DOI:** 10.1186/1475-2859-10-3

**Published:** 2011-01-27

**Authors:** Rahul Kumar, Kazuyuki Shimizu

**Affiliations:** 1 Department of Bioscience and Bioinformatics, Kyushu Institute of Technology, Iizuka, Fukuoka 820-8502, Japan; 2 Institute of Advanced Bioscience, Keio University, Tsuruoka, Yamagada 997-001, Japan; 3 Systems Biology Group, Department of Chemical and Biological Engineering, Chalmers University of Technology, Kemivägen 10, SE-412 96 Göteborg, Sweden

## Abstract

**Background:**

It is important to understand the cellular responses emanating from environmental perturbations to redesign the networks for practical applications. In particular, the carbon (C) metabolism, nitrogen (N) assimilation, and energy generation are by far important, where those are interconnected and integrated to maintain cellular integrity. In our previous study, we investigated the effect of C/N ratio on the metabolic regulation of *gdhA, glnL, glt B,D *mutants as well as wild type *Escherichia coli *(Kumar and Shimizu, MCF, 1-17, **9**:8,2010), where it was shown that the transcript levels of *cyoA *and *cydB *which encode the terminal oxidases, *fnr *and *fur *which encode global regulators were significantly up-regulated under N-limited condition as compared to C-limited condition. In the present study, therefore, the effects of such single-gene knockout on the metabolic regulation were investigated to clarify the roles of those genes in the aerobic continuous culture at the dilution rate of 0.2 h^-1^.

**Results:**

The specific glucose consumption rates and the specific CO_2 _production rates of *cyoA, cydB, fnr*, and *fur *mutants were all increased as compared to the wild type under both C-limited and N-limited conditions. The former phenomenon was consistent with the up-regulations of the transcript levels of *ptsG *and *ptsH*, which are consistent with down-regulations of *crp *and *mlc *genes. Moreover, the increase in the specific glucose consumption rate was also caused by up-regulations of the transcript levels of *pfkA*, *pykF *and possibly *zwf*, where those are consistent with the down regulations of *cra, crp *and *mlc *genes. Moreover, the transcript levels of *rpoN *together with *glnK, glnB, glnE *were up-regulated, and thus the transcript levels of *glnA,L,G*, and *gltB,D *as well as *nac *were up-regulated, while *gdhA *was down-regulated. This implies the interconnection between cAMP-Crp and P_II_-Ntr systems. Moreover, *cyoA*, *cydB*, *fnr *and *fur *gene deletions up-regulated the transcript levels of respiration (*nuoA, ndh, cyoA, cydB*, and *atpA*) and the oxidative stress related genes such as *soxR*, *S *and *sodA*, where this was further enhanced under N-limitation. In the cases of *cyoA *and *cydB *mutants, *arcA, fnr, fur, cydB *(for *cyoA *mutant), and *cyoA *(for *cydB *mutant) genes were up-regulated, which may be due to incomplete oxidation of quinol. It was also shown that *fur *gene transcript level was up-regulated in accordance with the activation of respiratory chain genes. It was shown that the deletion of *fur *gene activated the enterobactin pathway.

**Conclusion:**

The present result demonstrated how the fermentation characteristics could be explained by the transcript levels of metabolic pathway genes as well as global regulators in relation to the knockout of such single genes as *cyoA, cydB, fnr*, and *fur*, and clarified the complex gene network regulation in relation to glycolysis, TCA cycle, respiration, and N-regulated pathways. The present result is quite important in understanding the metabolic regulation for metabolic engineering. Moreover, the present result may be useful in improving the specific glucose consumption rate and activation of the TCA cycle by modulating the respiratory chain genes and the related global regulators. The result obtained under N-limited condition may be useful for the heterologous protein production under N-limitation.

## Background

**Figure 1:**
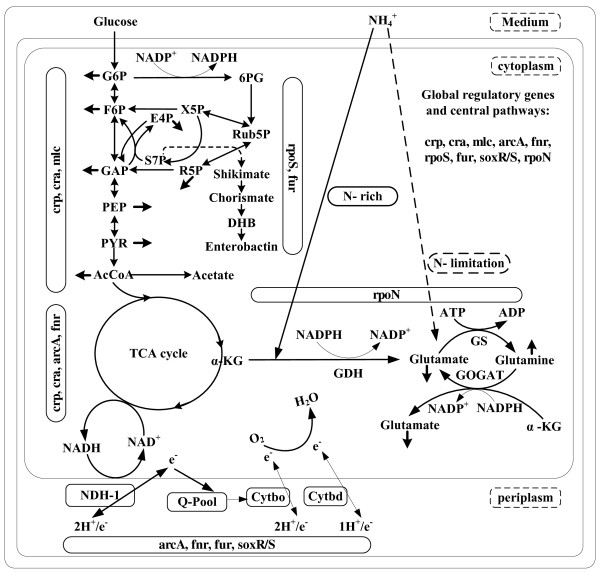
**Schematic diagram of integrated carbon, nitrogen, energy metabolism and its global regulation in *E. coli***.

It is important to understand the cellular responses emanating from the environmental perturbations to redesign the networks for practical applications as well as for theoretical studies [1-3]. Microorganisms such as *Escherichia coli *live in environments which are subject to rapid changes in the availability of carbon (C) and nitrogen (N) sources [4,5]. The carbon metabolism, nitrogen assimilation, and energy generation are integrated to maintain the cellular integrity (Figure 1). The limitation of such nutrients stimulates hunger state responses in bacteria, which turns the emphasis on scavenging substrates and induction for stimulating nutrient acquisition [6-8]. Microbes adapt to the low nutrient conditions by maintaining high metabolic fluxes that may reduce the energetic efficiency of overall metabolism [9,10]. In the case of N-assimilation, energy independent glutamate dehydrogenase (GDH) pathway is used when sufficient amount of nitrogen is present, while energy dependent glutamine synthetase-glutamate synthase (GS-GOGAT) pathway is used under N-limitation (Figure 1) [2,11,12]. The bulk of energy is generated by the respiratory chain in *E. coli *under aerobic condition, and its efficiency depends upon the cumulative activity of various elements [13-15]. The aerobic respiratory chain consists of multiple elements such as NADH dehydrogenases, catalyzing the generation of proton motive force during NADH oxidation, and quinone pool containing terminal oxidases, transferring electrons to oxygen (Figure 1). It is also crucial for the maintenance of redox balance [16,17].

In our previous study, metabolic regulations of *E. coli *and its single-gene knockout mutants such as *gdhA, glnL, gltB *and *gltD *mutants were investigated at various C/N ratios in the aerobic continuous culture [18]. From this study, it was found that transcript levels of the terminal oxidases encoded by such genes as *cyoA *and *cydB*, and the global regulatory genes such as *fnr *and *fur *were up-regulated under N-limitation especially at the C/N ratio of 16.48. The *cyoA *and *cydB *genes code for cytochrome *bo *oxidase, functional under aerobic condition (H^+^/e, 2), and cytochrome *bd*, active under microaerobic condition (H^+^/e, 1), respectively [19]. The *fnr *gene product, Fnr protein, is a primary transcriptional regulator that mediates the transition from aerobic to anaerobic growth [20-22]. The *fur *gene codes for the transcriptional activator Fur (Ferric uptake regulator), which controls its own synthesis as well as the transcription of genes involved in the iron homeostasis [23,24]. It also participates in the regulation of other cellular functions such as oxidative stress, glycolysis, TCA cycle, respiration, 2, 3-dihydroxybenzoate biosynthesis etc. [25-28]. From the practical application point of view, the role of aerobic respiratory chain and global regulators such as Fur has been highlighted in the biofuel production using *E. coli *[29-31]. The attention has also been paid to understand the role of Fur in iron uptake mechanisms with a view to find novel antimicrobial targets [32,33]. In the present study, therefore, the effects of *cyoA *and *cydB *genes knockout as well as *fnr *and *fur *genes knockout on the metabolism of *E. coli *were investigated based on fermentation characteristics and transcriptional mRNA levels in the aerobic continuous culture under both C- and N- limitations.

## Results

### Fermentation characteristics of the mutants

Table 1 shows the fermentation characteristics of *cyoA, cyd, fnr and fur *mutants for N- rich (C/N ratio 1.68), and N- limited (C/N ratio 8.42) conditions, respectively. It shows that the biomass concentrations of the mutants were reduced as compared to that of the wild type strain. In the case of *cyoA *mutant, the specific acetate production rate increased, while it decreased for the case of *cydB *mutant. Table 1 shows that the specific CO_2 _production rate was increased for both mutants as compared to that of the wild type under both N- rich and N- limited conditions. The specific glucose consumption rates of *cyoA *mutant were higher than those of the wild type for both N-rich and N-limited conditions. In the case of *fnr *mutant, biomass concentration reduced, and the specific acetate production rate and the specific glucose consumption rate increased under both N- rich and N- limited conditions as compared to those of the wild type (Table 1). The specific CO_2 _production rates were increased for the mutant as compared to those of the wild type under both N-rich and N-limited conditions. Similar to the case of *fnr *mutant, the biomass concentration reduced, while the specific acetate production rate increased for *fur *mutant under both N- rich and N- limited conditions (Table 1). One of the distinctive features of *fur *mutant was the pink color of fermentation broth under N-rich condition while it turned to violet under N-limitation [34].

**Table 1:** Fermentation characteristics of the wild type *E. coli*, and its *ΔcyoA*, *ΔcydB*, *Δfnr*, and *Δfur *mutants under aerobic continuous at dilution rate 0.2h^-^^1^: C/N ratios (a) 1.68, (b) 8.42. Biomass (g/l) indicates total cells in the fermentor, while cell yield (g/g) reflects biomass formed on the consumed glucose.

	Wild	*ΔcyoA*	*ΔcydB*	*Δfnr*	*Δfur*
(A) C- limitation (C/N ratio = 1.68)					

Biomass (g/l)	3.33 ± 0.17	2.86 ± 0.14	3.08 ± 0.15	2.38 ± 0.12	2.63 ± 0.13

Glucose (g/l)	ND*	ND*	ND*	ND*	ND*

Acetate (g/l)	1.73 ± 0.09	1.64 ± 0.08	1.25 ± 0.06	2.06 ± 0.10	1.81 ± 0.09

Cell yield (g/g)	0.330 ± 0.02	0.286 ± 0.01	0.308 ± 0.02	0.238 ± 0.01	0.263 ± 0.01

Specific glucose consumption rate (mmol/gdcw.h)	3.33 ± 0.17	3.88 ± 0.19	3.60 ± 0.18	4.66 ± 0.24	4.21 ± 0.21

Specific acetate production rate (mmol/gdcw.h)	1.73 ± 0.09	1.91 ± 0.10	1.35 ± 0.07	2.88 ± 0.14	2.29 ± 0.11

Specific CO_2 _production rate (mmol/gdcw.h)	6.74 ± 0.34	7.45 ± 0.37	7.99 ± 0.40	9.00 ± 0.45	8.05 ± 0.40

(B) N- limitation (C/N ratio = 8.42)					

Biomass (g/l)	1.67 ± 0.08	1.56 ± 0.08	1.62 ± 0.08	1.48 ± 0.07	1.40 ± 0.07

Glucose (g/l)	3.03 ± 0.15	3.01 ± 0.15	3.22 ± 0.16	3.59 ± 0.18	3.68 ± 0.18

Acetate (g/l)	1.41 ± 0.07	1.58 ± 0.08	1.13 ± 0.06	1.32 ± 0.07	1.62 ± 0.08

Cell yield (g/g)	0.240 ± 0.01	0.223 ± 0.01	0.239 ± 0.01	0.231 ± 0.01	0.221 ± 0.01

Specific glucose consumption rate (mmol/gdcw.h)	4.65 ± 0.23	4.98 ± 0.25	4.67 ± 0.13	4.80 ± 0.24	5.02 ± 0.25

Specific acetate production rate (mmol/gdcw.h)	2.82 ± 0.14	3.38 ± 0.17	2.33 ± 0.12	2.98 ± 0.15	3.86 ± 0.19

Specific CO_2 _production rate (mmol/gdcw.h)	8.25 ± 0.41	8.88 ± 0.44	8.47 ± 0.42	9.23 ± 0.46	8.39 ± 0.42

ND* = Not detectable

### *Gene transcript levels of cyoA *mutant

**Figure 2:**
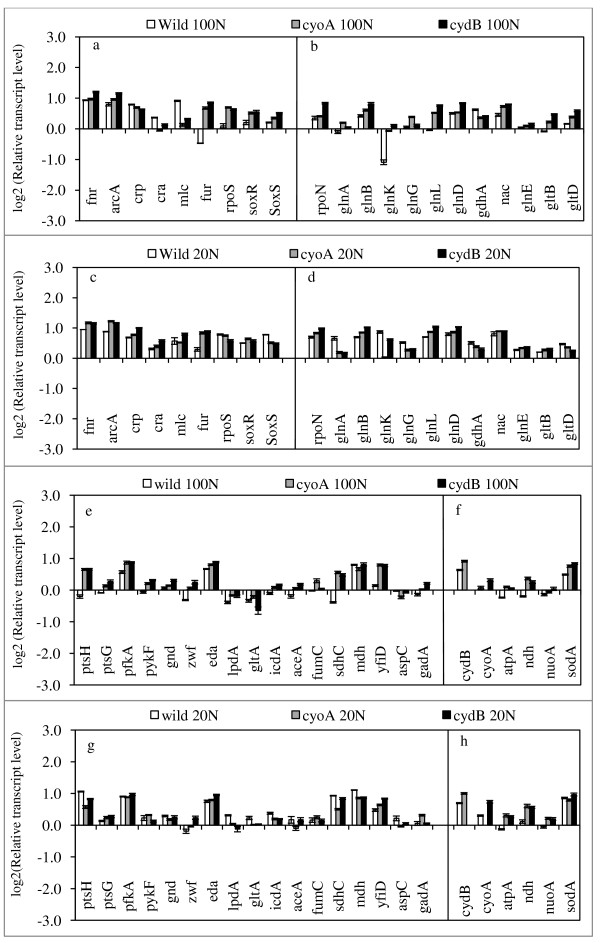
**Comparison of the transcriptional mRNA levels between the wild type *E. coli*, *cyoA *and *cydB *mutant genes at C/N ratio 1.68 and 8.42**.

The relative transcript levels for *cyoA *and *cydB *mutants are given as compared to those of the wild type in Figure 2, where it indicates that the transcript levels of *ptsG*, *ptsH*, *pfkA*, *pykF *as well as *zwf *and *eda *were all up-regulated (p < 0.01, p < 0.01, p < 0.01, p < 0.01; p < 0.01, p < 0.01, respectively) in accordance with the increase in the specific glucose consumption rate for *cyoA *mutant as compared to the wild type (Figure 2e). This is consistent with the down-regulations of the transcript levels of *cra *(p < 0.05), *crp *(p < 0.01), and *mlc *(p < 0.01) genes (Figure 2a) (see Additional file 1). Figure 2e also shows that the transcript levels of *lpdA*, *gltA*, *icdA*, *aceA*, *fumC*, and *sdhC *were increased (p < 0.01, p < 0.01, p < 0.01, p < 0.05, p < 0.01, and p < 0.01, respectively) for *cyoA *mutant as compared to the wild type under N- rich condition. The increased TCA cycle activity caused higher specific CO_2 _production rate for the mutant as compared to the wild type, and affected respiration. Figure 2f indicates that *cyoA *gene knockout caused the transcript level of *cydB *gene to be up-regulated, and those of other respiratory chain genes such as *nuoA*, *ndh*, and *atpA *genes were also up-regulated (p < 0.01, p < 0.01, and p < 0.01, respectively). This is consistent with the up-regulations of *soxR*, *S *and *sodA *(p < 0.01, p < 0.01, and p < 0.01). Moreover, the increase in *yfiD *may be due to increase of *arcA *(p < 0.01). Note that *aspC *was down regulated (p < 0.01) and *gadA *was up-regulated (p < 0.01), which will be discussed in the discussion section. Figure 2b indicates that the transcript level of *rpoN *increased, which caused the transcript levels of *glnB *and *glnK *to be up-regulated (p < 0.01 and p < 0.01), and those of *glnA*, *L*, *G *and *gltB*, *D *genes were up-regulated (p < 0.01, p < 0.01, p < 0.01, and p < 0.01, p < 0.01, respectively). Moreover, the transcript level of *nac *gene increased (p < 0.01) and *gdhA *gene decreased (p < 0.01). Those imply that GDH pathway was inativated, while GS pathway was activated for *cyoA *mutant (as well as *cydB *mutant) even under N-rich condition (see Additional file 2). This phenomenon was also enhanced under N-limited condition (Figure 2d). Note that *cyoA *knockout caused *rpoS *and *fur *transcript levels to be up-regulated (p < 0.01 and p < 0.01) while *fnr *transcript level changed little (Figure 2a).

Under N- limitation, the transcript level of *arcA *was up-regulated (p < 0.01), and this caused *lpdA *and TCA cycle genes such as *gltA*, *icdA*, *sdhC*, and *mdh *to be down-regulated (p < 0.01, p < 0.01, p < 0.01 and p < 0.01, respectively). The up-regulation of *arcA *also caused *yfiD *to be up-regulated (p < 0.01) for *cyoA *mutant as compared to wild type. Moreover, the respiratory chain genes such as *cydB*, *nuoA*, *ndh*, *atpA *were all up-regulated (p < 0.01, p < 0.01, p < 0.01, p < 0.01, respectively).

### Gene transcript levels of *cydB *mutant

Figure 2 also shows the transcript levels of *cydB *mutant as compared to those of the wild type and *cyoA *mutant. The transcript levels of nitrogen regulated genes were similar for both *cyoA *and *cydB *mutants. While *cydB *gene knockout caused the transcript level of *cyoA *to be up-regulated, the overall changing patterns of the transcript levels were quite similar to the case of *cyoA *mutant (Figure 2).

### Gene transcript levels of *fnr *mutant

**Figure 3:**
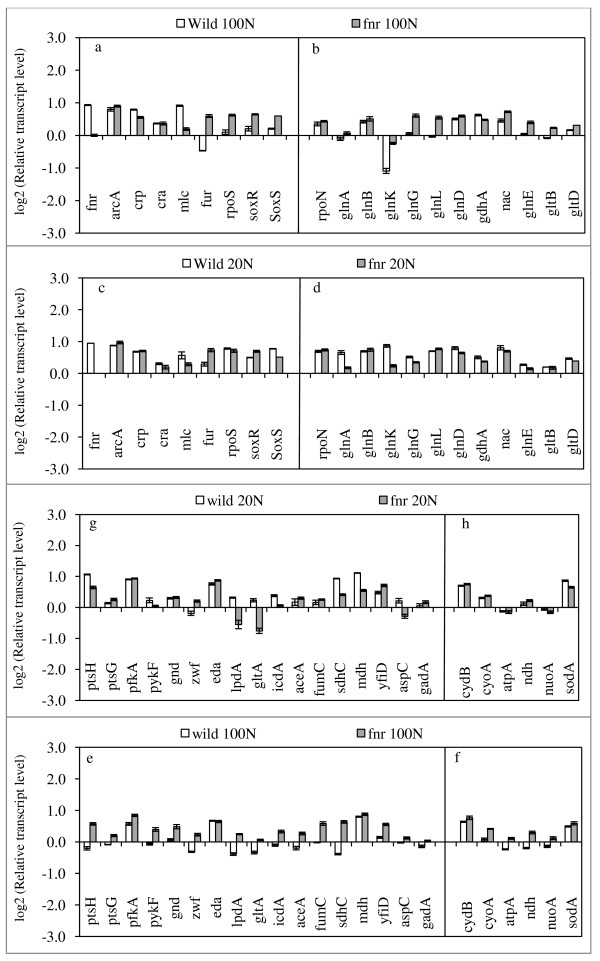
**Comparison of the transcriptional mRNA levels between the wild type *E. coli *and *fnr *mutant at C/N ratio 1.68 and 8.42**.

Figure 3 shows the effect of *fnr *gene knockout on the transcript levels, where it indicates that the transcript levels of the glucose uptake pathway and glycolysis genes such as *ptsG*, *ptsH*, *pfkA *as well as the oxidative PP pathway genes such as *zwf *and *gnd *were up-regulated for *fnr *mutant (p < 0.01, p < 0.01, p < 0.05; p < 0.01, and p < 0.05, respectively) in accordance with the increase of the specific glucose consumption rate as compared to the wild type under N- rich condition (Figure 3e). Some of those are consistent with the down regulation of the transcript levels of *crp *and *mlc *(p < 0.01 and p < 0.01) (Figure 3a). Although the transcript level of *arcA *changed little, the transcript levels of *lpdA *(p < 0.01) and TCA cycle genes such as *gltA*, *icdA*, *fumC*, *sdhC*, *mdh *as well as glyoxylate pathway gene *aceA *were up-regulated (p < 0.01, p < 0.01, p < 0.01, p < 0.01, p < 0.01; p < 0.01, respectively) for the mutant as compared to the wild type under N- rich condition (Figure 3e), which is consistent with the increased specific CO_2 _production rate for the mutant as compared to the wild type (Table 1). This is also consistent with up-regulations of *soxR*, *S *genes (Figure 3a). Figure 3b indicates that the transcript levels of *rpoN*, *glnB*, *glnK*, and *glnE *were up-regulated (p < 0.01, p < 0.01, p < 0.01, and p < 0.01 respectively), and those of *glnA*, *L*, *G*, and *gltB*, *D *were also up-regulated. The transcript level of *nac *gene was up-regulated (p < 0.01), while that of *gdhA *gene was down-regulated (p < 0.01) (Figure 3b). Those indicate that GDH pathway was inactivated, while GS pathway was activated even under N-rich condition (see Additional file 2). Note that the transcript levels of *rpoS *and *fur *genes were up-regulated for the mutant (Figure 3a).

Under N- limitation, the transcript levels of *ptsH *and *pykF *were decreased (p < 0.01 and p < 0.01) (Figure 3g), and those of *lpdA*, *gltA*, *sdhC*, and *mdh *were down-regulated for the mutant as compared to the wild type (p < 0.01, p < 0.01, p < 0.01 and p < 0.01, respectively). Moreover, the transcript levels of *glnA*, *glnG*, and *glnK *were decreased as compared to the wild type under N-limitation.

### Gene transcript levels of *fur *mutant

**Figure 4:**
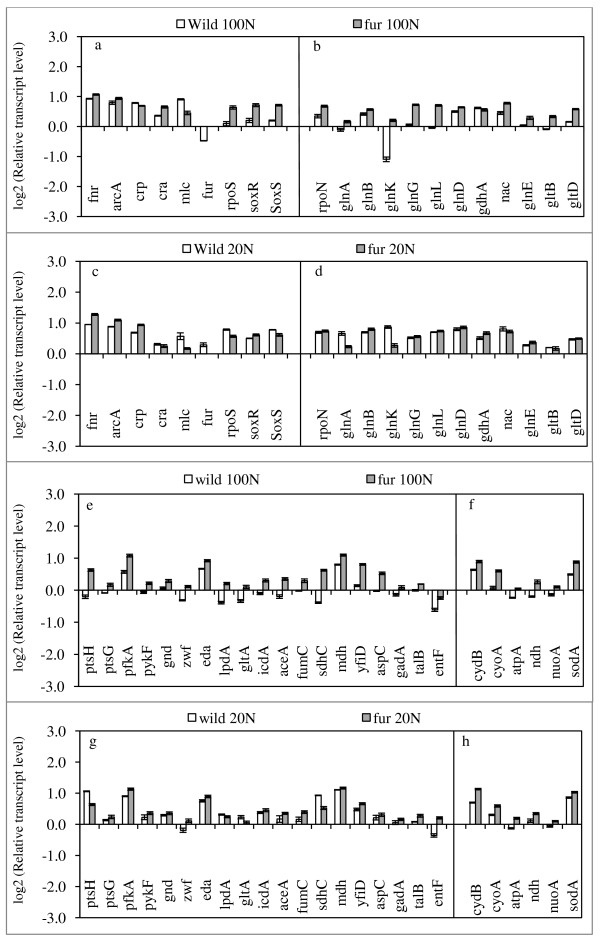
**Comparison of the transcriptional mRNA levels between the wild type *E. coli *and *fur *mutant at C/N ratio 1.68 and 8.42**.

Figure 4 shows the effect of *fur *gene knockout on the RNA transcript levels, where it indicates that the transcript levels of PTS genes such as *ptsH *and *ptsG*, and the glycolysis genes such as *pfkA *and *pykF *as well as the oxidative pentose phosphate (PP) pathway genes such as *zwf*, *gnd*, and the ED pathway gene *eda *were all up-regulated as compared to wild type under N- rich condition (p < 0.01, p < 0.01; p < 0.01, p < 0.01; p < 0.01, p < 0.01, and p < 0.01, respectively) (Figure 4e). This is consistent with the fermentation data where the specific glucose consumption rate was higher for the mutant as compared to the wild type (Table 1). The activation of TCA cycle genes is consistent with the activation of the respiration where the transcript levels of *cyoA*, *cydB*, *nuoA*, *ndh*, *atpA *as well as *sodA *genes were all up-regulated (p < 0.01, p < 0.01, p < 0.01, p < 0.01, p < 0.01, and p < 0.01, respectively) (Figure 4f). Moreover, the transcript level of *rpoN *increased (p < 0.01), and the transcript levels of *glnK*, *glnB*, *glnE *were up-regulated (p < 0.05, p < 0.01, p < 0.01), and those of *glnA*, *L*, *G*, and *gltB*, *D *genes as well as *nac *gene were up-regulated (p < 0.01, p < 0.01, p < 0.01, and p < 0.01, p < 0.01, respectively), while *gdhA *gene transcript expression was decreased (p < 0.01)(Figure 4b). Those imply that GDH pathway was inactivated, while GS pathway was activated even under N-rich condition as also seen by enzyme activities (see Additional file 2).

Under N- limitation, the transcript levels of *lpdA*, *gltA*, *fumC*, *sdhC*, and *aceA *were down-regulated (p < 0.05, p < 0.01, p < 0.01, p < 0.01 and p < 0.01, respectively), which may be partly due to the up-regulation of *arcA *(p < 0.01) (Figure 4c). The up-regulations of *arcA*, possibly caused the increase of the transcript level of c*ydB *(p < 0.01) (Figure 4h). Moreover, the respiratory chain genes such as *nuoA*, *ndh *and *atpA *were all up-regulated for the mutant as compared to the wild type (p < 0.01, p < 0.01 and p < 0.01, respectively) (Figure 4h). The transcript levels of *talB *and *entF *genes in *fur *mutant were up-regulated (p < 0.01, p < 0.01) under both N- rich and N- limited conditions as compared to the wild type (Figure 4e, g).

## Discussion

Figure 2 indicates that *cyoA *gene knockout caused the transcript level of *cydB *gene to be up-regulated, while *cydB *gene knockout caused *cyoA *transcript level to be up-regulated under both N- rich and N- limiting conditions. The aerobic respiratory chain of *E. coli *can function with either of the two different membrane-bound NADH dehydrogenase I (*nuo *operon) and NADH dehydrogenase II (*ndh*), where those generate quinones, and serve as important electron carriers for respiratory chain, where cytochrome bo_3 _(*cyoABCD*) and cytochrome bd (*cydAB*) utilize them for proton motive force. Malfunction of these would diminish the ability to convert NADH to NAD^+ ^and transfer electron to oxygen by converting quinone to quinol and generate proton motive force. Figure 2 also indicates that the transcript levels of *arcA *were up-regulated for both *cyoA *and *cydB *mutants. ArcA is activated through phosphorylation by a membrane protein ArcB, where this phosphorylation occurs after quinone inhibition of ArcB autophosphorylation is released [35]. Quinones are electron carriers where isoprenoid chain anchors them to the membrane, and those are thought to regulate the ArcB-ArcA two component system in response to redox state, which results in a release of quinone inhibition on ArcB, and subsequent autophosphorylation of ArcB and activation of *arcA*. Moreover, the increase in redox ratio NADH/NAD^+ ^, caused by the decreased NADH oxidation due to *cyoA *or *cydB *knockout and by the activation of TCA cycle implied by the specific CO_2 _production rate (Table 1), may have also activated the transcript levels of *arcA *gene [17,36]. The activation of *arcA *(or phosphorylated ArcA) may have caused *nuoA*, *ndh*, and *fumC *transcript levels to be up-regulated for *cyoA *and *cydB *mutants as compared to the wild type. In the case where removal of all of three cytochlome oxidases such as *cyo*, *cyd*, and *cbd *would result in anaerobic growth characteristics even under oxic conditions and may produce small amount of lactate for NADH reoxidation [37]. However, in the present study, *cydB *was active in *cyoA *mutant and *cyoA *was active in *cydB *mutant, and thus the quinol oxidase could have been worked, though NADH/NAD^+ ^ratio tended to increase, which may have caused *arcA *to be up-regulated. Moreover, NADH can be converted to NADPH by PntAB [38], and thus the cell tries to minimize the inbalance of NADH/NAD^+ ^ratio.

Figure 2c shows the up-regulation of *fnr *gene, where it has been known that the transcriptional regulator Fnr of *E. coli *functions as an O_2 _sensor, and the protein is in the active form and is predominately found as a homo-dimer with one [4Fe-4S] cluster per monomer under anoxic conditions. In the presence of oxygen, [4Fe-4S] Fnr is converted to [2Fe-2S] cluster and finally to apoFnr, which is no longer active in gene regulation [39,40]. Nevertheless, *fnr *gene transcript level increased for *cyoA *and *cydB *mutants, and *fnr *mutant gives some effect under N-limitation even under aerobic condition. This indicates that Fnr does not play its conventional role, but may have some role under aerobiosis.

It was previously shown that the specific glucose consumption rate as well as the specific CO_2 _production rate was up-regulated under N- limitation as compared to N-rich condition for the wild type [18]. The same phenomenon was observed in *cyoA *and *cydB *mutants even under N- rich condition. Part of the reason may be explained as follows: In the cultivation of *cyoA *and *cydB *mutants, cAMP-Crp was down-regulated, where this caused *mlc *gene to be down-regulated, which caused *ptsG *and *ptsH *genes to be up-regulated as seen in Figure 2e, where *ptsG *is also under control of ArcA. Note that the increase in glucose concentration causes cAMP to be decreased due to the increase in unphosphorylated E IIA^Glc^, which does not activate Cya. The expression of *crp *may be indirectly related with this. In the upstream of the *mlc *gene, there is a binding region of Crp, and *mlc *expression changed in accordance with *crp *gene expression [41]. Moreover, the increase in the glucose concentration caused *cra *to be down-regulated (Figure 2a), and caused *pfkA*, *pykF*, and *zwf *genes to be up-regulated (Figure 2e). Those together caused the specific glucose consumption rate to be up-regulated. Under N- limited condition, the specific growth rate and the transcript levels of glucose uptake, glycolysis, and the oxidative PP pathway genes were further up-regulated as compared to those of the wild type. As for N- regulation genes, *nac *was up-regulated, while *gdhA *gene was down-regulated for *cyoA *and *cydB *mutants as compared to the wild type under N- rich condition. Moreover, *glnB*, *K*, and *D *transcript levels were up-regulated, and *glnA*, *L*, *G *and *gltB*, *D *transcript levels were also up-regulated, which is consistent with the up-regulation of *rpoN*. This implies the interplay between cAMP-Crp and P_II_-Ntr systems between C metabolism and N assimilation, where it was shown that Crp can be recruited by sigma 54 holoenzyme (Eσ^54^) to a site upstream of *glnAp2 *[42].

When a particular nutrient becomes limiting, *E. coli *increases the production of proteins that forage for the limiting nutrient. Among the scavenging regulons, Crp, which allows the use of alternative carbon sources, and the two component NtrB/NtrC system, which controls scavenging for nitrogen are by far important in practice. Crp surveys carbon status with cAMP, while Ntr system surveys nitrogen status by glutamine. In the case of carbon catabolism in relation to glucose, the affinity to glucose may change depending on the glucose concentration in the fermentor. Under the culture condition of higher glucose concentration, the affinity of glucose is constant, where glucose forms complex with E IICB^Glc ^and phosphorylated by E IIA^Glc^-P. Under low glucose concentration such as shown in Table 1, where glucose concentration could not be detected, the affinity to glucose may change depending on the activities of Lam (in outer membrane) and MglBAC (in inner membrane) under such hunger state [43]. The transcript levels of *crp *as shown in Figure 2, 3, &4 may help estimating the relative glucose concentration. In the case of nitrogen regulation, the affinity of NH4 + is also different depending on the NH_4_^+ ^concentration. Namely, under N-limited condition (NH_4_^+ ^< 5 μM), AmtB is used with high affinity, while Amt is blocked by binding GlnK when NH_4_^+ ^> 50 μM [44], and its transport is made mainly by diffusion.

In the case of *fnr *mutant under N-rich condition, the similar mechanism may exists. Namely, the cell concentration decreased and the glucose concentration increased, which caused *crp *transcript level to be down-regulated and thus *mlc *gene was also down-regulated as state above (Figure 3a). This caused *ptsG *and *ptsH *to be up-regulated (Figure 3e). This, together with up-regulations of *pfkA *and *pykF*, caused the specific glucose consumption rate to be increased as compared to the wild type. The *fnr *gene deletion caused *cyoA *and *gltB,D *to be up-regulated (Figure 3b) (see Additional file 1), which were also caused by the down-regulation of *crp *transcript level (see Additional file 1). Loss of ability of an *fnr *or *narXL *mutant to reduce nitrate will result in more reduction of the ubiquinone pool, and hence repression of components of the ArcA regulon such as *cyoA*, and thus Fnr should indirectly activate rather than repress *cyoA*. This effect is compounded by the fact that *arcA *transcription is directly activated by Fnr, which in turn is essential for oxidation of ubiquinol by the cytoplasmic or periplasmic nitrate reductase, NarG or Nap [45]. The down-regulation of *crp *also caused g*lnA,L*, *aceA*, and *gltA *to be up-regulated (see Additional file 1). Those are consistent with the increased specific CO_2 _production rate. The reason why *gdhA *was repressed while *glnA,L,G, gltBD *were up-regulated may be due to increased *rpoN*, though not significant (Figure 3b).

In the case of *fur *gene knockout, *cyoA, sdhC*, and *sodA *genes transcript levels were up-regulated, where those genes are repressed by Fur (see Additional file 1). The increase in *rpoN *transcript level is consistent with the up-regulations of *nac, glnA,L,G, gltB,D *together with *glnB,K,E *and down-regulation of *gdhA*.

Under N-limitation, the up-regulation of *arcA *may have caused down-regulation of *lpdA *and *gltA *transcript levels (see Additional file 1). Those are also repressed in the media containing excess glucose known as glucose catabolite repression [36,25]. Under N- limitation, the increased glycolytic flux results in the increase of pyruvate, a substrate of PDH encoded by complex *pdhR-aceEF-lpdA *operon, that has been reported to induce *yfiD *gene [46]. The gene products of *yfiD *and *gadA *have been reported to confer oxidative and acid resistance, respectively [47,48]. Meanwhile, *aspC *transcript level decreased by the deletion of terminal oxidases under both N-rich and N- limited conditions as compared to the wild type, while it was up-regulated for *fur *mutant under both conditions. The *aspC *gene encodes aspartate aminotransferase (AspC) that synthesize aspartate and also provides alternate route to glutamate synthesis [49].

Moreover, consider why *cydB *gene transcript level was up-regulated for *cyoA*, *fnr*, *fur *mutants, and also for the wild type under N- limitation, where it has been reported to scavenge O_2 _under micro-aerobic condition [50]. Each cytochrome redox reaction is catalyzed by a low-spin heme which oxidizes quinon and feeds electrons to a highspin heme center that reduces O_2 _[51,52]. The *cydAB *operon is transcribed from a complex element containing total five promoters. These promoters P1, P2, P3, and P4 are regulated by O_2_, *arcA *and *fnr *gene products. The P5 promoter provides significant fraction of total *cydAB *transcription in aerobic environment [50,53].

The catabolic oxidation of electron carriers, which are mostly reduced flavoproteins, by molecular oxygen results in the production of reactive oxygen species that could damage DNA, lipid membranes, and proteins [54,55]. The cytotoxic effects of the reactive oxygen species are mostly mediated by iron, and the regulation of iron uptake has been reported to minimize oxidative stress induced by iron [56]. The aerobic respiratory chain activity appears to be changed little in *fnr *mutant as compared to the wild type under N- limitation. As opposed to the case of *fnr *mutant, the deletion of *fur *gene highly activates aerobic respiratory chain under N- limitation. It could be due to the de-repression of the iron uptake system which is repressed by Fur [57]. Although both *fnr *and *fur *genes appear to contribute to the oxidative stress regulation, the mechanisms may be different. It may be considered that *fnr *increases the synthesis of iron containing proteins, while *fur *regulates the iron uptake mechanism. The cellular regulations in *E. coli *are adapted to avoid toxicity from endogenous oxidants generated as a result of oxidative stress, where present study indicates that *fnr *and *fur *genes play important roles in these regulations. The effects of *fnr *and *fur *genes on oxidative stress were implied by the up-regulations of the transcript levels of *soxR, soxS *and *sodA *genes.

The metal levels are often sensed by metal-sensing regulatory RNA, which encodes metal-sensing proteins involved in the transport and storage of intra-cellular metals [58,59]. In the native environment, *E. coli *continuously faces iron deficiency which is one of the essential trace metals functioning as cofactor in many of the cellular constituents such as flavoproteins, and therefore, it is evolutionarily furnished with the mechanism to regulate iron uptake and storage system [54,57]. However, excessive iron may cause oxygen toxicity by catalyzing the formation of reactive free radicals through such reactions as Fenton/Haber-Weiss [55]. It has been known that aerobic respiration generates superoxide ions (O_2_^-^), with NDH II as the main generator of endogenous superoxide and NDH I and SDH as small contributors [60]. The malfunction of these enzymes would also decrease the O_2_^-^. Combination with inability to convert NADH to NAD^+^, a decrease in endogeneous O_2_^- ^would cause reductive stress, which may activate Fur [61]. Fur requires binding to Fe^2+ ^to become active. It was shown previously that O_2_^- ^deactivates Fur after its conversion to H_2_O_2 _by superoxide dismutase, through Fenton reaction [62] (H_2_O_2 _+ Fe^2+ ^→ HO^• ^+ OH^- ^+ Fe^3+^). Therefore, a decrease in endogeneous O_2_^- ^generation would increase the availability of Fe^2+^, through a decrease in H_2_O_2 _level, and in effect activate Fur relative to control [30]. Namely, Fur senses the reductive stress and represses genes in which Fe-S clusters are safe from damage by reactive oxygen species. It is essential for the cell to use iron economically, which is achieved to some extent by a combination of regulations such as siderophores synthesis, iron transport genes regulation etc. [56]. For these reasons, iron transport and siderophores (e.g. enterobactin) pathway related genes such as *talB, entF *are repressed by Fur protein [33,63,64]. In the present study, the transcript level of *fur *gene was up-regulated in particular under N- limitation, which may not be due to the iron deficiency by considering sufficient iron concentration in the medium. In the absence of *fur *gene, *E. coli *needs an alternate mechanism of iron homeostasis, and activated enterobactin pathway might have contributed to this regulation.

There are functional interactions between the carbon and ion utilization regulators Crp and Fur, and it was shown that the TCA cycle was repressed by the loss of both transcription factors [25]. In the present study, it was shown that TCA cycle was activated for *cyoA, cydB, fnr*, and *fur *mutants as compared to wild type under both C-limited and N-limited conditions. The *sdh *operon (*sdhCDAB*) encoding for subunits of iron-dependent SDH showed strong glucose repression, and strong apparent activation by Crp by both wild type and *fur *mutant [25]. The present result indicates that *fur *was significantly up-regulated in *cyoA, cydB*, and *fnr *mutants as compared to that of wild type, and *sdhC *was down-regulated (Figure 2, 3), while it was up-regulated in *fur *mutant (Figure 4).

**Figure 5:**
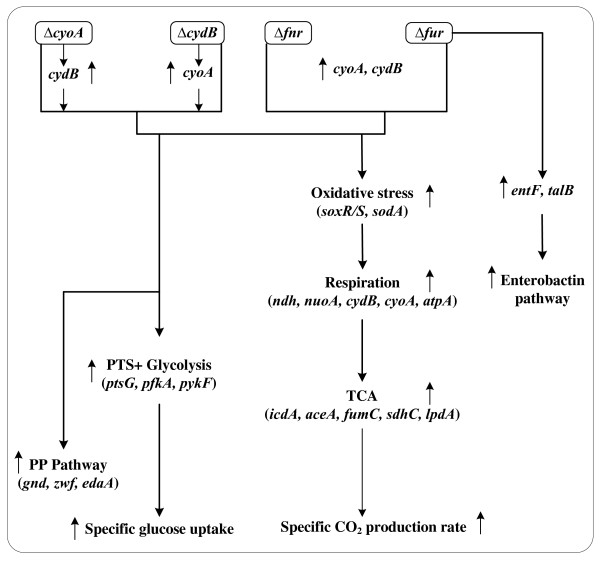
**Overall illustration for the effects of *cyoA, cydB, fnr*, and *fur *gene knockout on the metabolism based on the fermentation characteristics**.

**Figure 6:**
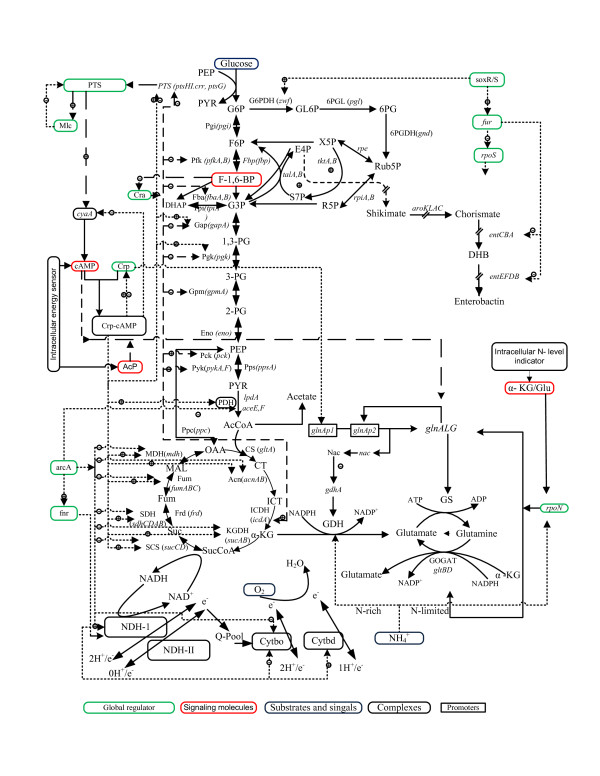
**Overall illustration of the gene regulation in terms of global regulators in central carbon metabolism of *E. coli***.

The overall fermentation characteristics were summarized in relation to glycolysis, TCA cycle, and respiratory chain genes in Figure 5. This can be interpreted in more detail by considering the effect of global regulators as shown in Figure 6.

## Conclusion

The present result demonstrated how the fermentation characteristics could be explained by the transcript levels of metabolic pathway genes as well as global regulators in relation to a single gene knockout such as *cyoA, cydB, fnr*, and *fur *mutants, and clarified the complex gene network regulation in relation to glycolysis, TCA cycle, respiration, and N-regulated pathway. The present result is quite important in understanding the metabolic regulation in relation to metabolic engineering. Moreover, the present result may be useful in improving the specific glucose consumption rate and activation of the TCA cycle by modulating the respiratory chain genes and the related global regulators. The result obtained under N-limited condition may be useful for the heterologous protein production under N-limitation.

## Materials and methods

### Strains, media composition, and cultivation conditions

The strains used were *Escherichia coli *BW25113 (F^- ^λ^- ^*rph-1 *Δ*araBADAH33 lacI^q ^*Δ*lac*Z_WJ16 _*rrn*B_T14 _Δ*rhaBAD*_LD78 _*hsd*R514), and its single gene knockout mutants lacking such genes as *cyoA *(JW0422), *cydB *(JW0723), *fnr *(JW1318) and *fur *(JW0669). These single gene knockout mutants were obtained from Keio collection [65]. All the strains were first precultured in the Luria-Bertani medium. The second preculture and the main culture were carried out using M9 minimal medium containing 10 g of glucose together with the following components (per liter): 6.81 g Na_2_HPO_4_, 2.99 g KH_2_PO_4_, 0.58 g NaCl and 5.94 g (NH_4_)_2_SO_4_. The following components were filter sterilized and then added (per liter) with 1 ml of 1 M MgSO_4_.7H_2_O, 1 ml of 0.1 mM CaCl_2 _2H_2_O, 1 ml of 1 mg/l thiamine HCl and 10 ml of trace element solution containing (per liter): 0.55 g CaCl_2_.2H_2_O, 1.67 g FeCl_3_.6H_2_O, 0.1 g MnCl_2_.4H_2_O, 0.17 g ZnCl_2_, 0.043 g CuCl_2_.2H_2_O, 0.06 g CoCl_2_.2H_2_O, and 0.06 g Na_2_MoO_4_.2H_2_O. The concentrations of nitrogen source such as (NH_4_)_2_SO_4 _were 1.188 g/l and 5.94 g/l, where the concentrations of all the other medium components were kept constant. The continuous culture was conducted in a 1-l fermenter (MDL 100, Marubishi Co., Tokyo, Japan) with a working volume of 500 ml. The pH was controlled at 7.0 ± 0.05 using 2 N HCl or 2 N NaOH, and the temperature was set at 37°C. The air flow rate was 1 vvm (air volume/working volume/min), and the agitation speed was 350 rpm to maintain the dissolved oxygen concentration at 35-40% (v/v) of air saturation [66]. The CO_2 _concentrations were monitored using an off-gas analyzer (BMJ-02 PI, ABLE Co., Japan). The dilution rate was 0.2 h^-1 ^for all the continuous cultures. The samples were collected at the steady state which was confirmed by the constant off-gas and cell density. It generally took 5-6 residence times to achieve the steady state.

### Analytical method

Bacterial growth was monitored by measuring the optical density of the culture broth at 600 nm (OD_600_) using a spectrophotometer (Ubet-30, Jasco, Tokyo, Japan). It was converted to dry cell weight (DCW) based on the OD_600_-DCW relationship previously obtained [67]. Glucose and acetate concentrations in the medium were measured using commercially available kits (Wako Co., Osaka, Japan for glucose; Roche, Molecular Biochemical, Mannheim, Germany for acetate).

### RNA preparation, design of PCR primers

Total RNA was isolated from *E. coli *cells by Qiagen RNeasy Mini Kit (QIAGEN K.K., Japan) according to the manufacturer's recommendation. The quantity and purity of the RNA were determined by the optical density measurements at 260 and 280 nm and by 1% formaldehyde agarose gel electrophoresis. The sequences of primers for respective genes used in this study were reported elsewhere [18,68], except such genes as *entF *and *talB*. The primer sequences of these additional genes are as follows:

*entF *TTTATTGCCGATCCTTTTGC (Left)

GGTAACGGCTTGTTCGACAT (Right)

*talB *CGTTTGTTGGCCGTATTCTT (Left)

AGAATTTCGCCGATGTTACG (Right)

Criteria for the design of the gene-specific primer pairs were followed according to *Molecular Cloning: A Laboratory Manual *[69]. The primers used in this study were synthesized at Hokkaido System Science Co. (Sapporo, Hokkaido, Japan). In all cases, the primer-supplied company confirmed the purity and absolute specificity of primers.

### c DNA synthesis and PCR amplification

RT-PCR reactions were carried out in a TaKaRa PCR Thermal Cycler (TaKaRa TP240, Japan) using Qiagen OneStep RT-PCR Kit (QIAGEN K.K., Japan). The reaction mixture was incubated for 30 min at 50°C for reverse transcription (cDNA synthesis) followed by 15 min incubation at 95°C for initial PCR activation. Then, the process was subjected to 30 cycles of amplification which consisted of a denaturing step (94°C for 1 min), an annealing step (approximately 5°C below melting temperature of primers for 1 min), and an extension step (72°C for 1 min), and then finally the reaction mixture was incubated for 10 min at 72°C for final extension. To check for nucleic acid contamination, one negative control was run in every round of RT-PCR. This control lacks the template RNA in order to detect possible contamination of the reaction components. 5 ml of amplified products were run on a 1% agarose gel. Gels were stained with 1 μg ml^-1 ^of ethidium bromide, photographed using a Digital Image Stocker (DS-30, FAS III, Toyobo, Osaka, Japan) under UV light and analyzed using Gel-Pro Analyzer 3.1 (Toyobo, Osaka, Japan) software. Although the PCR products obtained for all the genes showed the predicted sizes on agarose gel, the identity of amplified fragments of some genes was demonstrated by DNA sequencing. In order to determine the optimal amount of input RNA, the two-fold diluted template RNA was amplified in RT-PCR assays under identical reaction conditions to construct a standard curve for each gene product. When the optimal amount of input RNA was determined for each gene product, RTPCR was carried out under identical reaction conditions to detect differential transcript levels of genes. The gene *dnaA*, which encodes *E. coli *DnaA transcription dual regulator and is not subjected to variable expression, i.e. abundant expression at relatively constant rate in most cells, was used as an internal control for the RT-PCR determinations [68]. To calculate the standard deviation, RT-PCR was independently performed three times for each gene under identical reaction condition. To ensure that the observed changes were statistically significant, the Student's t-test was applied.

### Enzyme activities

The enzyme activities were measure for GDH, GS, and GOGAT, where assay methods are described elsewhere [18].

## Competing interests

The authors declare that they have no competing interests.

## Authors' contributions

RK carried out fermentation experiments, assayed, made statistical analysis, analyzed the results, and drafted the manuscript. KS considered the experimental design, analyzed the results, and prepared manuscript together with RK. All authors read and approved the final manuscript.

## Supplementary Material

Additional file 1 **Effect of global regulators on metabolic pathway related genes**. The table lists the known effects of global regulators on metabolic pathway related genes. Click here for file

Additional file 2 Enzyme Activity (Unit*/mg protein). The data shows the activities of the nitrogen assimilatory enzymes (GDH, GS, and GOGAT). Click here for file
