# Supplementary material for: Transcriptional regulation of main metabolic pathways of cyoA, cydB, fnr, and fur gene knockout Escherichia coli in C-limited and N-limited aerobic continuous cultures
Source: Microb Cell Fact. 2011 Jan 27;10:3. doi: 10.1186/1475-2859-10-3 (PMC3037301; doi:10.1186/1475-2859-10-3)
Supplement: Additional file 1 — Effect of global regulators on metabolic pathway related genes. The table lists the known effects of global regulators on metabolic pathway related genes. [file 1475-2859-10-3-S1.PDF]

Supplemental Data

S-1 Effect of global regulators on metabolic pathway related genes

Global Regulators	Regulation	Regulated Genes
Cra	+	*cydB, fbp, icdA, pckA, pgk, ppsA*
	-	*aceBAK, acnB, adhE, eda, edd, pfkA, pykF, zwf*
ArcA/B	+	*cydAB, focA, pflB*
	-	*aceBAK, aceEF, acnAB, cyoABCDE, fumAC, gltA, icdA, lpdA, mdh, nuoABCDEFGHIKLMN, pdhR, sdhCDAB, sodA, sucABCD*
Fnr	+	*acs, focA, frdABCD, pflB, yfiD*
	-	*acnAB, cyoABCDE, cydAB, fnr, fumA, icdA, ndh, nuoABCDEFGHIKLMN, sdhCDAB, sucABCD*
Mlc	+	
	-	*crr, manXYZ, malT, ptsG, ptsHI*
Crp/Cya	+	*acnAB, aceEF, focA, fumA, gltA, malT, manXYZ, mdh, mlc, pckA, pdhR, pflB, pgk, ptsG, sdhCDAB, sucABCD, ugpABCEQ*
	-	*cyaA, lpdA, rpoS*
SoxR/S	+	*acnA, fumC, fur, sodA, zwf*
	-	
RpoS	+	*acnA, acs, adhE, fumC, gadAB, osmC, poxB, talA, tktB,*
	-	*ompF*
Fur	+	
	-	*entABCDE, sodA,*

IclR	+	
	-	*aceBAK, acs*
PdhR	+	
	-	*aceEF, lpdA*
