# Supplementary material for: Transcriptional regulation of main metabolic pathways of cyoA, cydB, fnr, and fur gene knockout Escherichia coli in C-limited and N-limited aerobic continuous cultures
Source: Microb Cell Fact. 2011 Jan 27;10:3. doi: 10.1186/1475-2859-10-3 (PMC3037301; doi:10.1186/1475-2859-10-3)
Supplement: Additional file 2 — Enzyme Activity (Unit*/mg protein). The data shows the activities of the nitrogen assimilatory enzymes (GDH, GS, and GOGAT). [file 1475-2859-10-3-S2.PDF]

S-2: Enzyme Activity (Unit*/mg protein)

Enzyme	GDH		GS		GOGAT	
C/N ratio	1.68	8.42	1.68	8.42	1.68	8.42
Wild	0.269±0.013	0.137±0.005	0.105±0.001	0.303±0.016	0.022±0.006	0.047±0.002
Δ *cyoA*	0.473±0.035	0.162±0.014	0.099±0.001	0.310±0.040	0.029±0.001	0.022±0.001
Δ *cydB*	0.723±0.031	0.129±0.009	0.081±0.001	0.361±0.005	0.023±0.001	0.021±0.003
Δ *fnr*	0.258±0.053	0.131±0.005	0.087±0.008	0.357±0.007	0.021±0.001	0.018±0.003
Δ *fur*	0.604±0.035	0.247±0.014	0.111±0.014	0.317±0.041	0.034±0.002	0.043±0.004

*One unit is defined as the mM product formed in 1 min in 1 ml total reaction volume containing 0.1 ml sample volume.

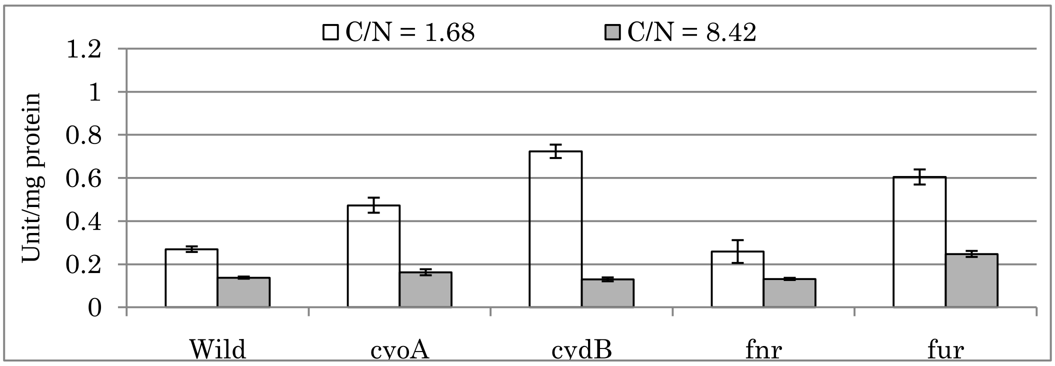

(a) GDH activity

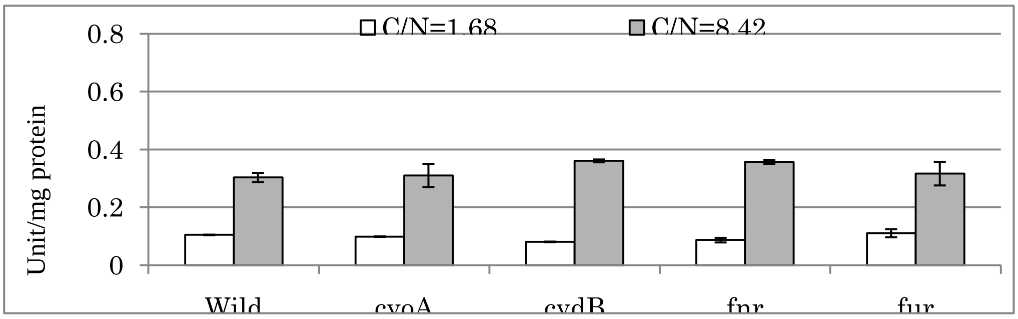

(b) GS activity

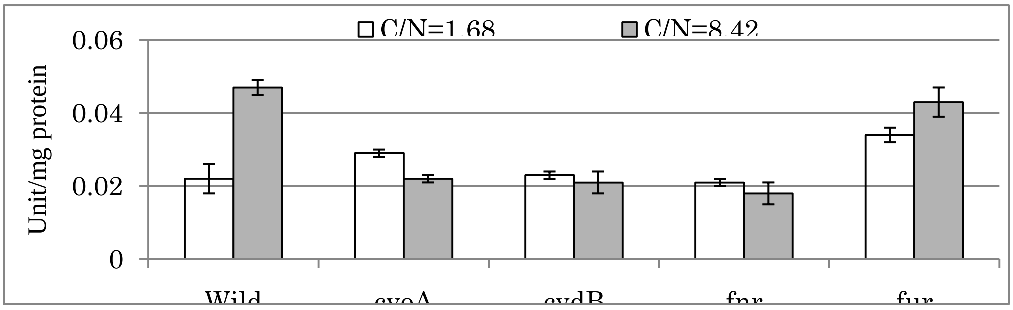

(c) GOGAT actibity
